# Development and optimization of sampling techniques for environmental samples from African swine fever virus-contaminated surfaces with no organic contaminants

**DOI:** 10.3389/fvets.2024.1425928

**Published:** 2024-07-18

**Authors:** Taeyong Kwon, Jordan T. Gebhardt, Eu Lim Lyoo, Natasha N. Gaudreault, Jessie D. Trujillo, Jason C. Woodworth, Chad B. Paulk, Cassandra K. Jones, Juergen A. Richt

**Affiliations:** ^1^Department of Diagnostic Medicine/Pathobiology, College of Veterinary Medicine, Kansas State University, Manhattan, KS, United States; ^2^Department of Animal Sciences and Industry, College of Agriculture, Kansas State University, Manhattan, KS, United States; ^3^Department of Grain Science and Industry, College of Agriculture, Kansas State University, Manhattan, KS, United States

**Keywords:** African swine fever, ASFV, environmental sample, fomite, surface

## Abstract

African swine fever (ASF) is a highly contagious diseases in domestic pigs and wild boars with up to 100% mortality. ASF virus (ASFV) is a causative agent responsible for ASF and highly resistant in environments, which creates a significant challenge for the control and eradication of the virus. Despite the geographical expansion of ASFV and international movement of products to sustain the swine production system, there is limited knowledge on the use of environmental samples to perform surveillance to prevent the introduction of ASFV into ASFV-free areas and for control of transmission in affected areas. Therefore, this study aimed to develop and optimize sampling techniques for environmental samples for ASFV detection. The stainless steel surfaces were contaminated with ASFV-infected blood, swabbed using different devices, and then processed through different techniques. The environmental samples were processed and tested using qPCR analysis. The results showed that the use of pre-moistened gauze surgical sponges, sweeping pads, and sponge sticks resulted in increased sensitivity, when compared to either dry sampling devices or Dacron swab. In particular, the combination of the sponge stick and the commercial nucleic acid preservative supported the best detection of ASFV DNA on the clean stainless steel surfaces evaluated. Pre-incubation for the short period of time and centrifugation at low speed were sufficient to provide satisfactory diagnostic sensitivity of ASFV detection using qPCR for environmental samples. Our findings contribute to the development of techniques for environmental samples for ASFV surveillance to prevent the introduction and dissemination of ASFV.

## 1 Introduction

African swine fever is a WOAH-reportable disease and fatal disease with up to 100% mortality in domestic pigs and wild boars. African swine fever virus (ASFV) is a causative agent and only member of family Asfaviridae. It is a double-stranded DNA virus, and its genome size is 150–180 kbs in length, encoding 150–180 proteins (1). Since its first identification in Kenya in the early 1900's, the virus exists in the wild in Africa and is maintained in the sylvatic cycle where the virus is transmitted between ticks and warthogs (2). In addition, modes of ASFV transmission to domestic pigs include direct contact to infected pigs or biological vectors, the consumption of ASFV-contaminated meat and/or carcasses and the exposure to ASFV-containing fomites. After the introduction of the genotype II ASFV into the Caucasian region (3), possibly through swill feedings in 2007, wild boars have been responsible for spreading ASFV gradually to Europe over the decade (4). Once the virus was first detected in China in 2018, the virus spread rapidly to the neighboring Asian countries. Given the geographical expansion in the short period of time, ASFV transmission could be mediated by human activities including the movement of humans, live domestic pigs, and/or fomites (5). In 2021, ASFV was detected in the Dominican Republic and spread to nearly all provinces in the country. Eventually, the virus was officially confirmed in the neighboring country, Haiti (6). This epidemiological situation poses a major threat to the US swine production system given the close geographical proximity to the mainland of North America.

Early detection is crucial to prevent the introduction and further spread of ASFV. The most common type of diagnostic samples includes animal-origin samples, such as whole blood, serum, swab and tissues, thus, the protocol for these types of samples has been well-established to ensure high sensitivity and specificity (7, 8). In contrast, despite the prolonged persistence in environments and the potential role of fomites in ASFV transmission (9), our knowledge of environmental samples remains still limited. Therefore, this study aimed to develop and optimize the sampling and processing of the environmental samples for better understanding prevention of ASFV introduction and control of ASFV transmission.

## 2 Materials and methods

### 2.1 Virus

The Georgia07 strain of ASFV was used in this study. Whole blood was collected from an experimentally infected pig in a previous animal study using an ETDA tube and stored at −80°C until the experiment was conducted. The virus titer of EDTA blood was 1.36 × 10^8^ TCID_50_/mL.

### 2.2 Experiment 1

A total of 16 drops of EDTA blood were added onto 10 × 10 cm stainless steel surfaces and dried for 30 min. Each drop was 6.25 μL and total inoculum was 100 μL per each surface. Each sampling strategy was applied to a separate stainless steel coupon, each with no organic material present, with a total of 5 replications for each sampling strategy. In total, eight different sampling strategies were tested: (1) 10 × 10 cm of dry cotton gauze (Dynarex Corporation, Orangeburg, NY, USA), (2) 10 × 10 cm of wet cotton gauze premoistened with 5 mL of phosphate buffered saline (PBS), (3) 10 × 10 cm of dry sweeping pad (Swiffer, P&G, OH, USA), (4) 10 × 10 cm of wet sweeping pad premoistened with 7.5 mL of PBS, (5) sponge stick containing 10 mL neutralizing buffer (Cat. #SSL10NB, 3M, St. Paul, MN, USA), (6) sponge stick (Cat. #SSL100, 3M, MN, USA) premoistened with 10 mL DNA/RNA shield (Zymo Research, Irvine, CA), (7) dry Dacron swab (Puritan Medical Products, Guilford, ME, USA), and (8) wet Dacron swab premoistened with 2 mL of PBS. A disposable tweezer was used to hold the cotton gauze and sweeping pad when swabbing. After swabbing on surface, the cotton gauze and the sweeping pad were placed in the 50 mL conical tube, the sponge stick in a sample bag, and the Dacron swab in a 2 mL cryovial. PBS was added into the tubes; 25 mL for (1) dry cotton gauze, 20 mL for (2) wet cotton gauze, 25 mL for (3) dry sweeping pad, 17.5 mL for (4) wet sweeping pad, and 2 mL for (7) dry Dacron swab. PBS volume for pre-moistening and elution was determined based on our routine sampling strategy for cotton gauze and sweeping pad (10) and Dacron swab (11), or manufacturers' instruction for the sponge sticks. After the tube was vortexed or the bag was massaged for 15 s, the supernatant was transferred into a new cryovial. The equal volume of supernatant and AL lysis buffer (Qiagen, Germantown, MD, USA) was mixed and stored at −80°C until further experiment.

### 2.3 Experiment 2

Experiment 2 aimed to compare different premoistened sampling devices in ASFV detection in environmental samples. The triplicates of ASFV-contaminated stainless steel surface for each sampling strategy were prepared as described in experiment 1. In experiment 2.1, five different sampling devices were tested: (1) the wet cotton gauze premoistened with 5 mL PBS, (2) the wet sweeping pad premoistened with 7.5 mL of PBS (3) the sponge stick containing 10 mL neutralizing buffer, (4) the sponge stick premoistened with 10 mL DNA/RNA shield, and (5) the wet Dacron swab premoistened with 2 mL of PBS. After placing in the tube or bag, PBS was added to the cotton gauze, the sweeping pad and the Dacron swab to normalize the total volume of liquid to 10 mL. After the tube was vortexed or the bag was massaged for 15 s, the supernatant was transferred into a new cryovial. The equal volume of supernatant and AL lysis buffer was mixed and stored at −80°C until further experiment. A 100 microliter of blood was mixed with 9.9 mL of PBS for positive control. PBS was used for negative control. In experiment 2.2, four different sampling approaches were evaluated for swabbing the ASFV-contaminated surface: (1) the wet cotton gauze premoistened with 5 mL PBS, (2) the wet cotton gauze premoistened with 5 mL DNA/RNA shield, (3) the sponge stick premoistened with 10 mL DNA/RNA shield, and (4) the sponge stick premoistened with 10 mL PBS. After placing the cotton gauze in the tube, either 5 mL PBS or DNA/RNA shield was added to the tube. The sponge stick was placed in the sample bag. The tube and bag were vortexed and massaged, respectively, for 15 s, and supernatant was transferred into the new cryovial. The AL lysate was generated as mentioned above and stored at −80°C until further experiment.

### 2.4 Experiment 3

In experiment 3, we determined the effect of pre-incubation on ASFV DNA in environmental samples. The triplicates of ASFV-contaminated surface for each pre-incubation condition were prepared and swabbed using (1) the wet cotton gauze premoistened with 5 mL PBS and (2) the sponge stick premoistened with 10 mL DNA/RNA shield as mentioned above. The gauze was placed in the 50 mL conical tube, and PBS was added to the tube. The stick was placed in the bag. The swab samples were incubated for (1) 5 min at room temperature (RT), (2) 1 h at RT, (3) 1 h at 4°C, (4) 24 h at RT, and (5) 24 h at 4°C. After incubation, the swab samples were vortexed or massaged, and supernatant was collected to make the AL lysate, which was stored at −80°C until further experiment.

### 2.5 Experiment 4

In order to determine the effect of centrifugation and filtration on ASFV DNA detection on environmental samples from stainless steel surface. For experiment 4, similar procedures were used as previously evaluated which included the inoculation of the stainless steel coupon with the mixture of 100 μL of ASFV-infected blood and 5 mL of PBS and dried for 30 min. The steel surface was swabbed by using the wet cotton gauze premoistened with 5 mL PBS, the gauze was placed in the 50 mL conical tube, and 5 mL of PBS was added to the tube. After vortexing for 15 s, the supernatant was aliquoted into three tubes, and each tube was subjected to no centrifugation, centrifugation for 5 min at 700 × g for 5, 10, and 15 min, or centrifugation at 10,000 × g for 5, 10, and 15 min. After the centrifugation, the supernatant was transferred into the new cryovial, and the AL lysate was generated and stored at −80°C until further experiment. Next, the swab samples were generated as mentioned previously: swabbed the contaminated steel surface with 100 μL blood and 5 mL PBS, and added 5 mL PBS into tube, and vortexed for 15 s and aliquoted into four microtubes. Each tube was subjected to (1) no processing, (2) centrifugation for 5 min at 700 × *g*, (3) filtration using a 0.45 μm syringe filter, or (4) centrifugation for 5 min at 700 × *g* and then filtration through 0.45 μm syringe filter. After treatment, the supernatant was collected to make the AL lysate and stored at −80°C until further experiment.

### 2.6 Quantitative PCR

ASFV DNA was extracted from the AL lysate using a magnetic-based extraction system as described previously (12). Briefly, the AL lysate was heat-inactivated at 70°C for 10 min, and 200 μL of the lysate and 200 μL of isopropyl alcohol were added into a pre-loaded extraction plate, and extraction was performed on the automatic extractor. Extracted DNA was mixed with the forward and reverse primers, probe, and PerfeCTa^®^ FastMix^®^ II (Quanta Biosciences; Gaithersburg, MD, USA), in a total of 20 μL reaction. PCR reaction was performed on CFX machine. The Cq value was converted to copy numbers/mL using the standard curve.

### 2.7 Statistical analysis

ASFV DNA copy number was log-transformed and analyzed in GraphPad Prism 10 (GraphPad Software, San Diego, CA, USA). For experiment 1, *t*-tests were performed to compare dry and wet device conditions. Analysis of variance was used for experiments 2, 3, and 4. Within all statistical analysis, the positive control treatment was excluded.

## 3 Results

**Figure 1:**
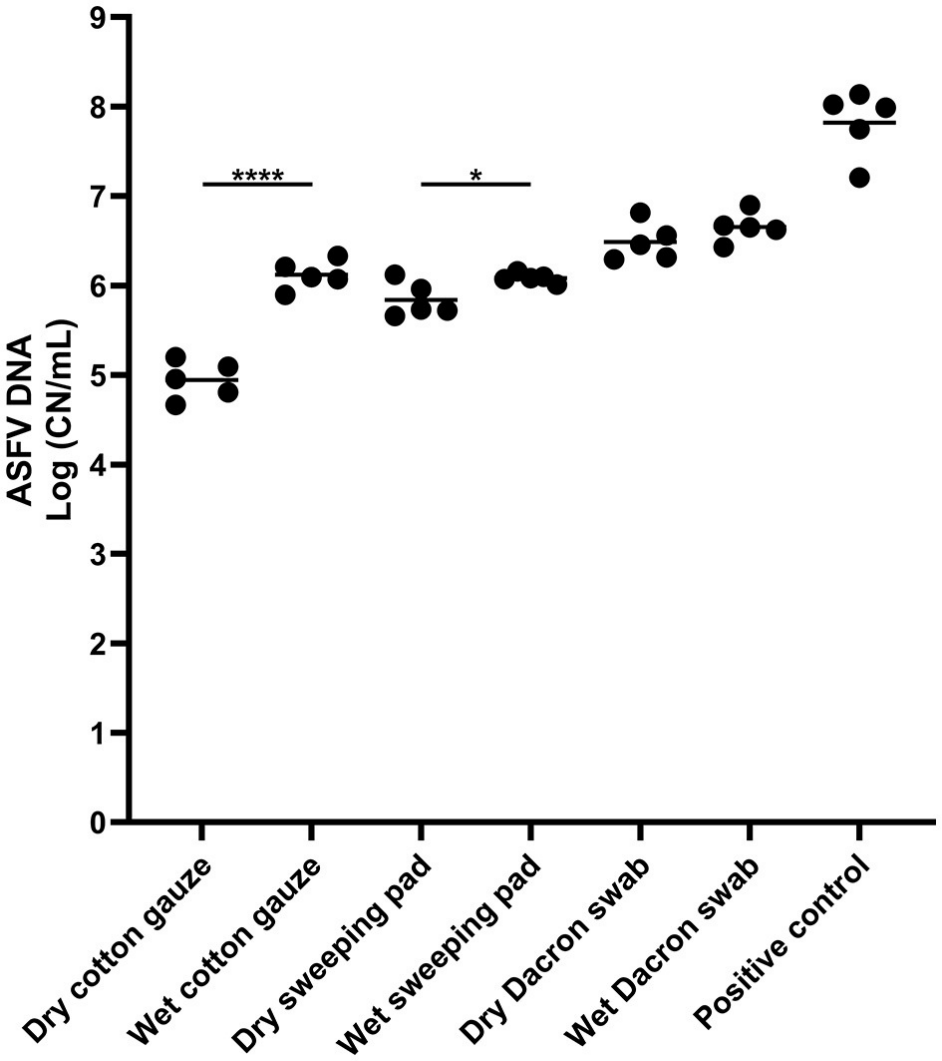
African swine fever virus (ASFV) DNA detection in environmental samples. Stainless steel surfaces were inoculated with 100 μL of ASFV-infected blood and swabbed using different types of sampling devices. The supernatant was subjected to quantitative PCR detecting ASFV DNA. A 100 microliter of blood was used for positive control. The amount of ASFV DNA (copy numbers per mL) was log-transformed for statistical analysis and the central tendency was represented mean of log-transformed values. Statistical differences between each dry and wet devices were assessed by Student *t*-test (*p*-value <0.05: * and <0.0001: ****).

To compare the dry and wet sampling devices for ASFV detection, three different samples devices were used to swab ASFV-contaminated surfaces and supernatant was tested for ASFV detection by qPCR. All samples tested were positive for ASFV detection (Figure 1). The highest ASFV detection was found in the samples from the dry and wet Dacron swabs, and there was no significant difference in ASFV detection between dry and wet swabs. In contrast, we found significantly higher detection of ASFV in wet cotton gauze and sweeping pad when compared to dry sampling devices, respectively.

**Figure 2:**
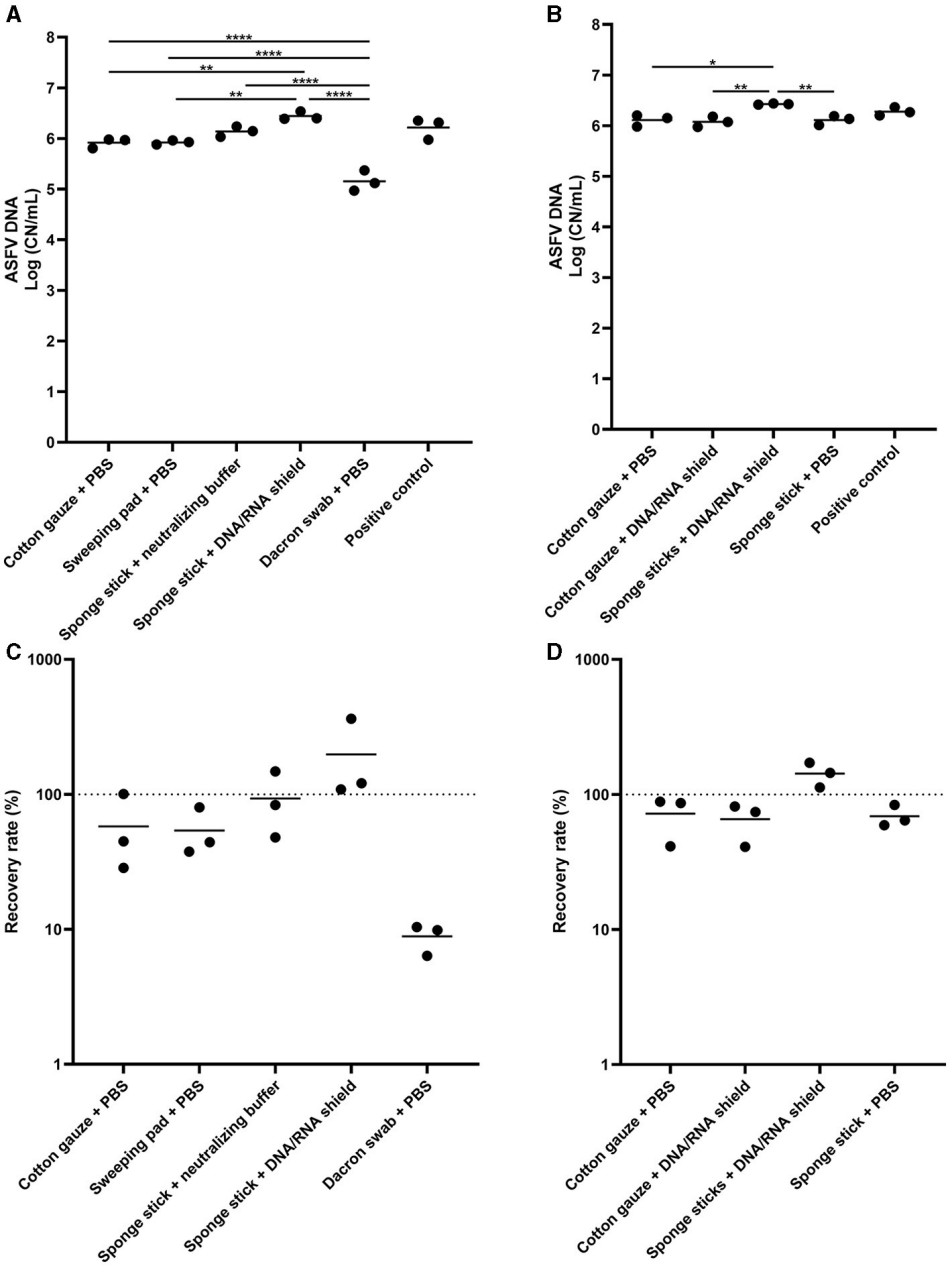
African swine fever virus (ASFV) DNA detection in environmental samples. Stainless steel surfaces were inoculated with 100 μL of ASFV-infected blood and swabbed using five different types of sampling devices **(A, C)** or four combinations of devices and buffers **(B, D)**. After normalization to 10 mL, the supernatant was subjected to quantitative PCR detecting ASFV DNA. Positive control was 100 of μL blood in 10 mL of PBS. The amount of ASFV DNA (copy numbers per mL) was log-transformed for statistical analysis and the central tendency was represented mean of log-transformed values **(A, B)**. Recovery rate (%) was calculated by dividing the amount of ASFV DNA of the sample by that of the positive control and central tendency was represented mean **(C, D)**. Statistical differences among sampling devices were assessed by ANOVA (*p*-value <0.05: *, <0.01: **, and <0.0001: ****).

Next, we tested the several sampling devices and different buffers for pre-moistening devices. In the first experiment, we compared the five different conditions and normalized to 10 mL of the buffer. The highest ASFV DNA detection was found in the sponge stick pre-moistened with DNA/RNA shield, and the amount of DNA was significantly higher than those in wet cotton gauze, wet sweeping pad, and wet Dacron swab (Figure 2A). In the samples of the Dacron swabs, we found the lowest ASFV DNA detection. Two sampling devices, cotton gauze and sponge stick, and two buffers, PBS and DNA/RNA shield, were selected for further analysis and a total of four different combinations of sampling devices were tested. We observed the highest virus detection when the ASFV-contaminated surfaces were swabbed using the sponge stick containing DNA/RNA shield (Figures 2B–D).

**Figure 3:**
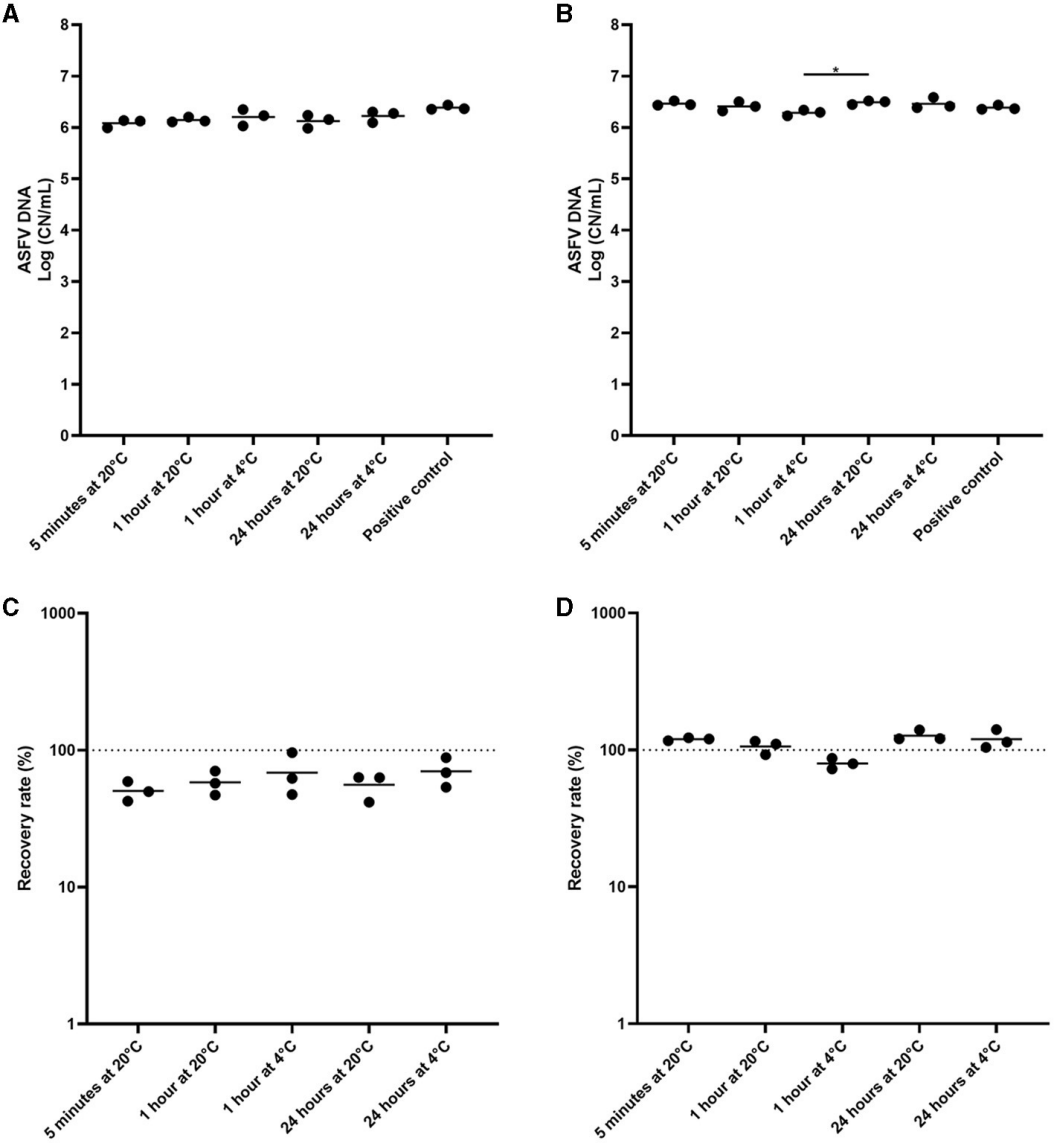
Comparison of pre-incubation conditions of environmental samples from African swine fever virus (ASFV)-contaminated surfaces for ASFV detection. Stainless steel surfaces were inoculated with 100 μL of ASFV-infected blood, swabbed using the cotton gauze premoistened with PBS **(A, C)** or the sponge stick with DNA/RNA shield **(B, D)**, and incubated at 4°C or room temperature (RT) for different time periods. After normalization to 10 mL, the supernatant was subjected to quantitative PCR detecting ASFV DNA. Positive control was 100 of μL blood in 10 mL of PBS. The amount of ASFV DNA (copy numbers per mL) was log-transformed for statistical analysis and the central tendency was represented mean of log-transformed values **(A, B)**. Recovery rate (%) was calculated by dividing the amount of ASFV DNA of the sample by that of the positive control and central tendency was represented mean **(C, D)**. Statistical differences among sampling devices were assessed by ANOVA (*p*-value <0.05: *).

In experiment 3, we incubated the environmental samples under different conditions to determine the most effective pre-extraction incubation conditions prior to analysis by qPCR. The environmental samples were prepared and incubated under five different conditions. There was no significant difference when cotton gauze was used (Figure 3A). In the sponge stick, the detection of ASFV DNA was similar across all conditions evaluated, with the exception of a small increase in detection when incubating samples at 24 h at RT compared to 1 h at 4°C (Figures 3B–D).

**Figure 4:**
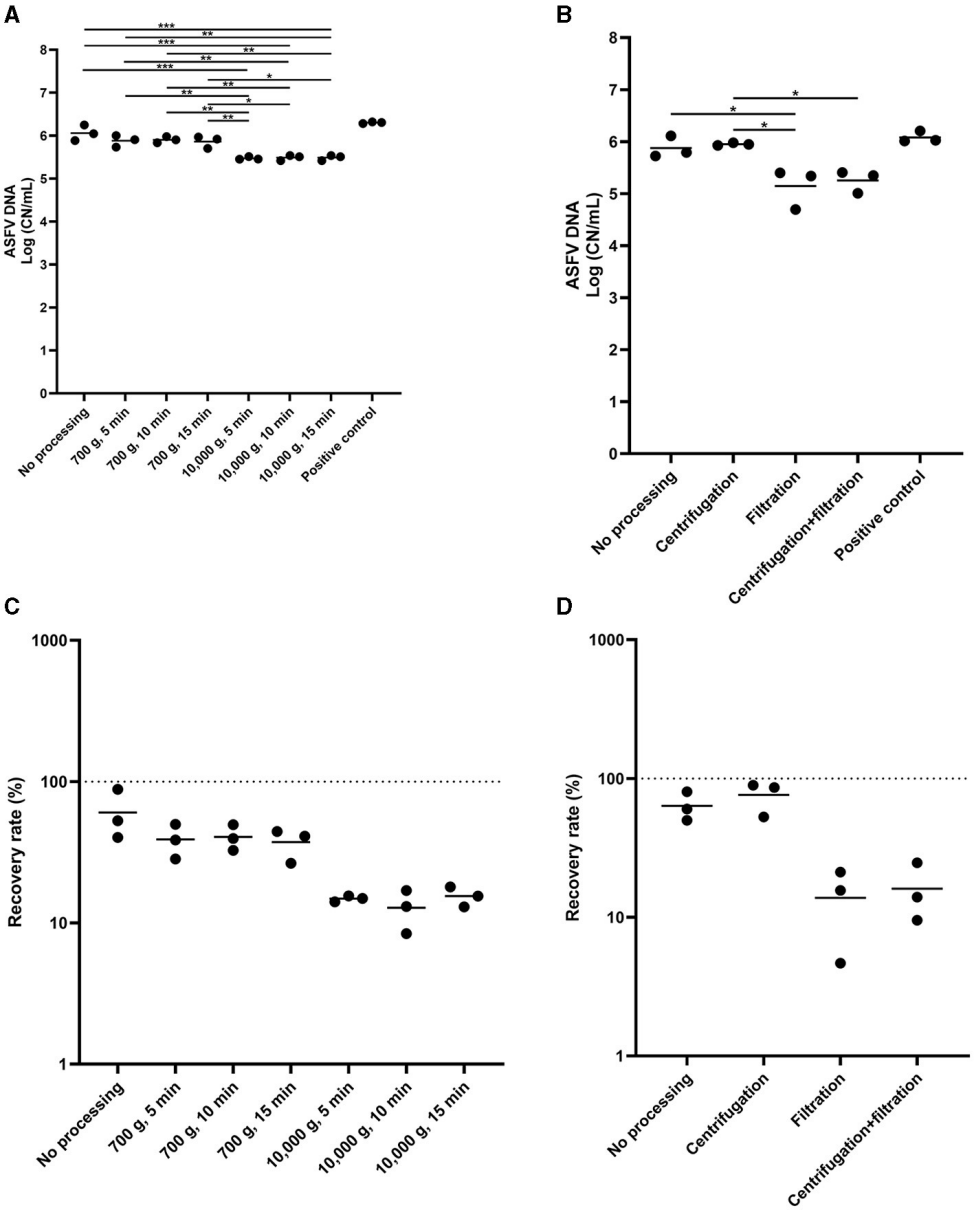
The effect of centrifugation and filtration on African swine fever virus (ASFV) detection in environmental samples. Stainless steel surfaces were inoculated with the mixture of 100 μL of ASFV-infected blood and 5 mL of PBS and swabbed using the cotton gauze premoistened with PBS. After normalization to 10 mL, the supernatant was aliquoted into several tubes and subjected to centrifugation **(A, C)** or combination of centrifugation and filtration **(B, D)** for ASFV detection. Positive control was 100 of μL blood in 10 mL of PBS. The amount of ASFV DNA (copy numbers per mL) was log-transformed for statistical analysis and the central tendency was represented mean of log-transformed values **(A, B)**. Recovery rate (%) was calculated by dividing the amount of ASFV DNA of the sample by that of the positive control and central tendency was represented mean **(C, D)**. Statistical differences among sampling devices were assessed by ANOVA (*p*-value <0.05: *, <0.01: **, and <0.001: ***).

Lastly, we determined the effect of centrifugation and filtration of the environmental samples on ASFV DNA detection. The low-speed centrifugation did not affect ASFV detection in the environmental samples (Figure 4A). In contrast, we found a significant reduction in the level of ASFV DNA after centrifugation at 10,000 × g. Furthermore, filtration had a negative impact on ASFV DNA detection in the environmental samples (Figures 4B–D).

## 4 Discussion

Because there is no available commercial vaccine against ASFV in domestic pigs and wild boars, the current strategy to control and prevent ASF outbreaks relies on biosecurity at individual farm level as well international boundaries to prevent introduction. Biosecurity involves restricting the movement of anything potentially causing disease, such as humans, live animals, animal products and/or fomites and eliminating them. One of the key points in ASFV preparedness and response is early detection of ASFV, which enables rapid detection and isolation of the site or object to prevent further spread to other areas. In contrast to the biological fluids or tissue samples that are common sample types for clinical diagnostic testing, environmental samples contain a variety of PCR inhibitors, resulting in the reduction of PCR sensitivity and false-negative results. In addition, despite the extended survival of ASFV in environments, the processing techniques of environmental samples guaranteeing high sensitivity have not been evaluated. Therefore, this study aimed to develop and establish the protocol for testing the environmental samples from the common surface, stainless steel, while not contaminated with any organic material such as dirt or fecal material.

First, we found that wet sampling devices resulted in better detection of ASFV DNA in the environmental samples. This result was consistent with the general protocol of microbiology for environmental sampling (13), in which moisture from either surfaces or sampling devices is required for effective sampling of surfaces. However, it should be noted that all samples from dry devices were also positive for ASFV detection. In some scenarios, immediate actions might be required to response and control urgent situations, when the appropriate reagents are not prepared in the field. Under this situation, dry sampling devices could be an alternative for environmental sampling to prevent potential cross-contamination in the preparation of sampling devices.

Next, we tested several sampling devices from practical devices which can be easily accessible in the field to specialized sampling tools. Sterile synthetic fiber swabs with plastic shafts have been commonly used to collect environmental samples from surfaces, however, the lowest recovery rate was identified in this study. This is because the limited contact area of the swab was not sufficient to transfer the contaminants from large surfaces used in this study. While these sampling devices are considered the industry standard for nasal, oropharyngeal, and rectal swabs or samples from tissues, the limited contact area limits their utility as environmental sampling devices. In contrast, ASFV detection was satisfactory in the environment samples using two practical devices containing the common buffer; cotton gauze and sweeping pad pre-moistened with PBS. This technique has been widely used to detect various bacterial and viral diseases in environmental samples in pig industries because of its easy accessibility and cost-effectiveness (14, 15). In particular, the materials have been successfully used to determine the level of ASFV contamination within a feed manufacturing and swine production system in the regions of active ASFV circulation as well as under the experimental conditions (10, 16–18). The highest detection was identified in the sponge stick with DNA/RNA shield, therefore, we decided to further determine the best combination of the sampling device and buffer. Interestingly, the significance was found only in the combination of the sponge stick and commercial nucleic acid preservative, implying the synergetic effect of them on ASFV detection in the environmental samples. The sponge stick is widely used in environmental microbiology because it is able to sample larger surface areas than standard swabs, giving more chances to capture microorganisms, and it contains the variety of buffers in the product for subsequent enrichment of bacteria (19). This efficient capacity of transferring contaminant to the testing samples might contribute to better detection of ASFV DNA in the presence of the DNA preservative, which would prevent the degradation of ASFV in the environment. A recent study showed that the use of the sponge sticks pre-hydrated with a surfactant liquid also resulted in similar sensitivity, when compared to the traditional sampling method using a cotton swab, with effective viral inactivation (20). In this study, the virucidal effect of the commercial nucleic acid preservative on environmental samples from ASFV-contaminated surfaces has not been evaluated, but other studies showed the efficient inactivation of several different viruses (21–23). Given that ASFV is not only a WOAH-reportable disease but also a select agent in US, the combination of the sponge stick and DNA/RNA shield could be one of the sampling methods ensuring the high sensitivity of ASFV detection without risk of potential exposure of ASFV samples during transportation and sample processing.

The key point of environmental sampling is to remove the contaminants from the surface and then release them from the sampling devices to the buffer for subsequent cultivation or quantitative analysis. Several different techniques allow the efficient release of contaminants to the buffer: incubation overnight in elution buffer (24), mechanical mixing using a vortex mixer, shaking with beads, ultrasonication, and/or stomaching (13). Given its biosecurity level, we excluded the methods that have potential risk of spills in the processing of samples and compared the different incubation conditions for better ASFV DNA detection. Our results showed that incubation for the short period of time at RT and subsequent vortexing was sufficient to release viral DNA from the sampling devices. It is worth noting that immediate responses are crucial to control and prevent ASF outbreaks, thus, this shortened sample processing technique would contribute to early detection of ASFV.

Lastly, one of the important findings in this study was that centrifugation at high speed and filtration reduced the sensitivity of ASFV detection in environmental samples. In contrast, low speed centrifugation of the environmental samples had no impact on ASFV DNA detection. These results were consistent with the previous findings in which centrifugation at low speed had no impact on diagnostic sensitivity for porcine epidemic diarrhea virus, but the reduced sensitivity was found after filtration (25). In this study, the effect of centrifugation and filtration on environmental samples containing organic matters was not evaluated, but it would improve our understanding of ASFV diagnostics for environmental samples which mimic the real-world situation where organic matters are present.

In the present study, the sampling devices and processing techniques for environmental samples have been evaluated for diagnostic purposes. The use of pre-moistened gauzes, sweeping pads and sponge sticks resulted in the greatest sensitivity. In particular, the combination of the sponge stick and the commercial nucleic acid preservative supported the best detection of ASFV DNA from clean stainless steel surfaces. Pre-incubation for the short period of time and centrifugation at low speed were sufficient to provide the sensitivity of ASFV qPCR for environmental samples under the conditions of this study.

## Data availability statement

The original contributions presented in the study are included in the article/supplementary material, further inquiries can be directed to the corresponding authors.

## Author contributions

TK: Conceptualization, Formal analysis, Investigation, Methodology, Writing – original draft, Writing – review & editing. JG: Conceptualization, Funding acquisition, Methodology, Project administration, Supervision, Writing – review & editing. EL: Formal analysis, Investigation, Writing – review & editing. NG: Funding acquisition, Methodology, Writing – review & editing. JT: Investigation, Methodology, Writing – review & editing. JW: Funding acquisition, Writing – review & editing. CP: Funding acquisition, Writing – review & editing. CJ: Funding acquisition, Writing – review & editing. JR: Conceptualization, Funding acquisition, Methodology, Supervision, Writing – review & editing.
